# Royal Jelly Inhibits* Pseudomonas aeruginosa* Adherence and Reduces Excessive Inflammatory Responses in Human Epithelial Cells

**DOI:** 10.1155/2017/3191752

**Published:** 2017-09-17

**Authors:** Heni Susilowati, Keiji Murakami, Hiromichi Yumoto, Takashi Amoh, Kouji Hirao, Katsuhiko Hirota, Takashi Matsuo, Yoichiro Miyake

**Affiliations:** ^1^Department of Oral Biology, Faculty of Dentistry, Gadjah Mada University, Jl. Denta, Sekip Utara, Yogyakarta 55281, Indonesia; ^2^Department of Oral Microbiology, Institute of Biomedical Sciences, Tokushima University Graduate School, 3-18-15 Kuramoto-cho, Tokushima 770-8504, Japan; ^3^Department of Conservative Dentistry, Institute of Biomedical Sciences, Tokushima University Graduate School, 3-18-15 Kuramoto-cho, Tokushima 770-8504, Japan

## Abstract

*Pseudomonas aeruginosa* is a Gram-negative bacterium and causes respiratory infection especially in elderly patients. Royal jelly has been used worldwide as a traditional remedy and as a nutrient; however, the effect against* P. aeruginosa* is unclear. The aim of this study was to analyze antibacterial, antiadherent, and anti-inflammatory effects of royal jelly against* P. aeruginosa*. Wild-type strain PAO1 and clinical isolates of* P. aeruginosa* were used for antibacterial assay and antiadherent assay to abiotic surface and epithelial cells, which are pharynx (Detroit 562) and lung (NCI-H292) epithelial cells. In anti-inflammatory assay, epithelial cells were pretreated with royal jelly before bacterial exposure to investigate its inhibitory effect on interleukin (IL-8) and macrophage inflammatory protein-3*α*/CCL20 overproduction. Although royal jelly did not have antibacterial activity at concentration of 50% w/v, antiadherent activity was confirmed on the abiotic surface and epithelial cells under concentration of 25%. Pretreatment with royal jelly significantly inhibited overproduction of IL-8 and CCL20 from both cells. These results demonstrated that royal jelly inhibits* P. aeruginosa* adherence and protects epithelial cells from excessive inflammatory responses against* P. aeruginosa* infection. Our findings suggested that royal jelly may be a useful supplement as complementary and alternative medicine for preventing respiratory infection caused by* P. aeruginosa*.

## 1. Introduction

*Pseudomonas aeruginosa* is an opportunistic pathogen in human and is a Gram-negative rod which is abundantly found in soil, plant, decaying matter, and water. This bacterium is able to survive in various habitats, because it can use various organic compounds as carbon and nitrogen resources. Although it is classified as an obligate aerobe, this species can grow in anaerobic environment such as in periapical lesion caused by dental infection [1, 2]. In chronic periapical lesion,* P. aeruginosa* was identified in mixed population together with other species [2]. It was also one of frequent microorganisms which survive in persistent or secondary periapical infection and root canal tissue [3, 4]. It sometimes causes respiratory infection especially in elderly patients. Our previous report showed that* P. aeruginosa* colonized in oral cavity could be a risk factor of aspiration pneumonia in the elderly patients with cerebrovascular diseases and dysphagia [5]. It has also been known that* P. aeruginosa* is the most important etiological factor causing fatal nosocomial infections and found to be resistant to several antibiotics. Previous study has reported that this pathogen was identified as the second rank of species isolated from monomicrobial nosocomial bloodstream infection and caused 47.9% and 27.6% of mortality in intensive-care unit (ICU) patients and in non-ICU patients, respectively [6]. Some isolates were resistant to piperacillin, ticarcillin-clavulanate (Tic-Clv), ceftazidime, imipenem (IPM), aztreonam, ciprofloxacin (CPFX), gentamycin, and tobramycin [6]. The increasing of antibiotic resistant* P. aeruginosa* has become a worldwide problem.

Bacterial adherence to the surface of epithelial cells is an initial step in bacterial colonization and induction of pathological responses on host tissue [7]. Virulence factor molecules determine the ability of* P. aeruginosa* to induce pathological responses. Those factors also play important roles in bacterial colonization, survival, and their invasion into host tissue [8].

Royal jelly is a secretion produced from the hypopharyngeal and mandibular glands of young worker honeybees and contains all the nutrients to develop the queen honeybee from the larva. It is composed of water, carbohydrate, lipids, proteins, vitamins (mainly riboflavin, niacin, and thiamin), some minerals (mainly calcium and iron), and other components and has been used worldwide as a traditional and ethopharmacological nutrient and remedy [9]. A number of studies demonstrated that it possesses antimicrobial activities and antitumor and anti-inflammatory activities. However, the effect of royal jelly against* P. aeruginosa* was studied only to a limited extent and the results do not allow drawing definitive conclusions [10–12].

We made a hypothesis that increased adherence correlates to an increase in the production of proinflammatory cytokines and the concentrations of royal jelly inhibiting adherence are also inhibiting inflammation. Therefore, the aim of this study was to analyze the effect of royal jelly on the adherence of* P. aeruginosa* to abiotic surface and human pharyngeal and lung epithelial cell lines, Detroit 562 and NCI-H292, as good experimental model for* in vitro P. aeruginosa* adherence, and to examine cytotoxicity and anti-inflammatory effects of royal jelly on these human epithelial cells stimulated with* P. aeruginosa*.

## 2. Materials and Methods

### 2.1. Bacterial Strains, Growth Condition, and Antibiotics

*P. aeruginosa* PAO1, wild-type strain, and four clinical isolates, TUH-54, TUH-124, TUH-188, and TUH-213, were used in this study. Four clinical isolates were isolated from oral cavity or respiratory tract. IPM and CPFX were purchased from Wako Pure Chemical Industries (Osaka, Japan). Amikacin (AMK) was purchased from Sigma-Aldrich (St. Louis, MO, USA). All* P. aeruginosa* strains were grown at 37°C in lysogeny broth (LB) or on LB agar plates. For each experiment, bacterial cells were picked up from single colony, inoculated in LB broth, and incubated at 37°C for 16 h of shaking.

### 2.2. Cell Line Culture

Detroit 562 (American Type culture collection; ATCC, Manassas, VA, USA) and NCI-H292 (ATCC, Manassas, VA, USA) epithelial cell lines derived from pharynx and lung, respectively, were used. Detroit 562 cells were cultured in minimum essential medium alpha supplemented with 2 mM glutamine, 1% nonessential amino acids, 1 mM sodium pyruvate, 0.1% lactalbumin hydrolysate, 10% (vol/vol) fetal bovine serum (FBS), 100 *μ*g mL^−1^ streptomycin, and 100 units mL^−1^ penicillin, and NCI-H292 cells were cultured in RPMI1640 medium supplemented with 2 mM glutamine, 10% (vol/vol) FBS, 100 *μ*g mL^−1^ streptomycin, and 100 units mL^−1^ penicillin, in a water-saturated atmosphere of 95% air and 5% CO_2_ at 37°C. Both cells in medium were seeded in wells of 24-well tissue culture plate and incubated until confluent monolayers developed. Confluent monolayers were used in all experiments.

### 2.3. Royal Jelly Preparation

Royal jelly was purchased from Yamada Bee Farm (Okayama, Japan). Royal jelly was suspended in phosphate buffered saline (PBS) and stirred overnight at 4°C. The suspension was then centrifuged at 12,000*g* for 15 min at 4°C followed by filtration using 0.45 *μ*m pore filter and kept at 4°C until just before use. For bacterial susceptibility test, fresh working solution of royal jelly was prepared in PBS or LB broth.

### 2.4. Susceptibility Assay

The minimum inhibitory concentrations (MICs) of antibiotics and royal jelly were assessed by the standard microbroth dilution method. Approximately 1 × 10^6^ cells mL^−1^ of bacterial culture was inoculated into 100 *μ*l of LB broth containing a twofold serial dilution of antibiotics or royal jelly suspension in 96-well culture plate (TPP, Trasadingen, Switzerland) and incubated for 24 h at 37°C. The MIC was defined as the lowest concentration showing no bacterial growth.

### 2.5. Bacterial Adherence Assay

Bacterial adherence assay for abiotic surface was performed using 96-well plates. Royal jelly was added in the culture medium before bacterial inoculation at the concentration of 25%. Approximately 1 × 10^6^ cells mL^−1^ of* P. aeruginosa* culture was inoculated into 100 *μ*l of LB broth and then incubated for 6 h at 37°C. After incubation, adherent bacteria were washed with purified water twice without disturbing the adherent bacteria and stained with 0.1% crystal violet for 10 min at room temperature, and excess stain was removed by gentle washes with purified water twice. After being dried, stained adherent bacteria were extracted from well by adding 150 *μ*l of ethanol and the absorbance of the extract from stained adherent bacteria was measured at 595 nm using a microplate reader (model 680; Bio-Rad Laboratories, Hercules, CA, USA). For dose-dependent study, PAO1 and representative clinical isolate TUH-54 were cultured with 5, 12.5, 20, and 25% royal jelly suspension in culture medium.

Bacterial adherence assay for epithelial cells was also performed. Confluent Detroit 562 and NCI-H292 cell monolayers were preincubated with the various concentrations of royal jelly (12.5, 20, and 25%) for 30 min at 37°C. And then,* P. aeruginosa* PAO1 or TUH-54 was directly added to each epithelial cell in 24-well tissue culture plates at final concentration of 1 × 10^8^ cells mL^−1^ and incubated for 1 h. As a positive control, both epithelial cells were stimulated with bacteria without pretreatment of royal jelly. All experiments were done using antibiotic-free culture medium.* P. aeruginosa* adherence to royal jelly-pretreated epithelial cells was quantified by determining the number of colony-forming units (CFUs) after washing with PBS twice. Adherent bacterial cells from epithelial cells were isolated by lysis with water prior to spreading on LB agar plates. We confirmed that there is no effect of osmotic shock caused by pure water on the bacterial viability (data not shown). The plates were incubated at 37°C for 24 h and CFU counts of adherent* P. aeruginosa* were determined and adherence was expressed as the percentage of bacteria recovered from cell lysis to those of the initial inoculums.

### 2.6. Lactate Dehydrogenase (LDH) Cytotoxicity Assay

The effect of royal jelly on cell cytotoxicity was determined using LDH assay. Confluent Detroit 562 and NCI-H292 cell monolayers in 24-well plates were cultured with the various concentrations of royal jelly (5, 12.5, 20, and 25%) for 4 h at 37°C in a water-saturated atmosphere of 95% air and 5% CO_2_. As a positive control, the cells were treated with 0.1% Triton X-100 and gently shaken for 10 min at room temperature. For the cytotoxicity assay, the levels of LDH in the recovered cell culture supernatants were determined using LDH cytotoxicity assay kit (Cayman Chemical, Ann Arbor, MI, USA) in accordance with the manufacturer's instructions. Absorbance was measured at 490 nm using a microplate reader (Bio-Rad Laboratories).

### 2.7. Royal Jelly Protection Assay

Detroit 562 monolayers cultured in a 24-well plate were pretreated with 25% royal jelly or PBS for 30 s at room temperature, washed with PBS twice, and irritated with 0.1% Triton X-100 for 30 sec at room temperature. The levels of LDH in the recovered cell culture supernatants were determined using LDH cytotoxicity assay kit and absorbance was measured at 490 nm using a microplate reader.

### 2.8. Chemokine Stimulation Assay

Confluent monolayers of Detroit 562 and NCI-H292 cells were preincubated with various concentrations of royal jelly (5, 12.5, 20, and 25%) in antibiotic-free culture medium for 30 min at 37°C. After 30 min incubation, the cells were washed with PBS twice. And then* P. aeruginosa* PAO1 or TUH-54 was directly added to each epithelial cell in 24-well tissue culture plates at final concentration of 2.0 × 10^7^ cells mL^−1^ and incubated for 4 h. After 4 h incubations, the culture medium was collected and stored at −20°C until being assayed. Total RNA from the epithelial cells was isolated with NucleoSpin RNA II (MACHEREY-NAGEL, Duren, Germany).

### 2.9. Enzyme-Linked Immunosorbent Assay (ELISA)

ELISA kits (R&D Systems, Minneapolis, MN) were used to quantify IL-8 and CCL20 in cell culture supernatants collected after* P. aeruginosa* infection.

### 2.10. Reverse-Transcription-Polymerase Chain Reaction (RT-PCR)

RT and PCR were performed in two steps as follows. cDNA synthesis was performed with an RNA PCR Kit (TaKaRa, Shiga, Japan) and specific gene transcripts were amplified with ReddyMix PCR Mix (ABgene, Surrey, UK). The primers and PCR conditions for amplification of IL-8, CCL20, and glyceraldehydes-3-phosphate dehydrogenase (GAPDH) mRNA were described previously [13]. GAPDH was used as an internal control. PCR products were analyzed by agarose gel electrophoresis and ethidium bromide staining.

### 2.11. Statistical Analysis

All experiments were conducted in triplicate or quadruplicate and statistical analyses were performed using the multifactorial one-way analysis of variance (ANOVA) with Tukey's Multiple Comparison test. Differences were considered significant when probability values were less than 1% (*p* < 0.01).

## 3. Results and Discussion

### 3.1. Studying the Comparative Susceptibility of* P. aeruginosa* to Antibiotics and Royal Jelly

The antibacterial activities of antibiotics and royal jelly against* P. aeruginosa* were shown in Table 1. According to the Clinical and Laboratory Standard Institute guideline M100-S22 break-point, TUH-54 exhibited resistance to IPM. Royal jelly did not exhibit antibacterial activities (≤50% w/v) against all tested bacteria. Our results are consistent with results reported by Boukraa stating that four kinds of royal jelly had antimicrobial activities against* P. aeruginosa*; however, the MICs were from 60 to 100% [14]. From these results, we conclude that the royal jelly may have quite low antimicrobial activity against* P. aeruginosa*. Major Royal Jelly Protein-1 (MRJP-1), which is one of components in beehive product that is Jelleine glycoproteins, has been shown to inhibit the growth of multidrug resistant* P. aeruginosa* [15]. Regarding the potency of royal jelly, our research then was focused on investigating antiadhesion potential and protective function of royal jelly on host cells.

### 3.2. The Effect of Royal Jelly on the Attachment of* P. aeruginosa*

**Figure 1:**
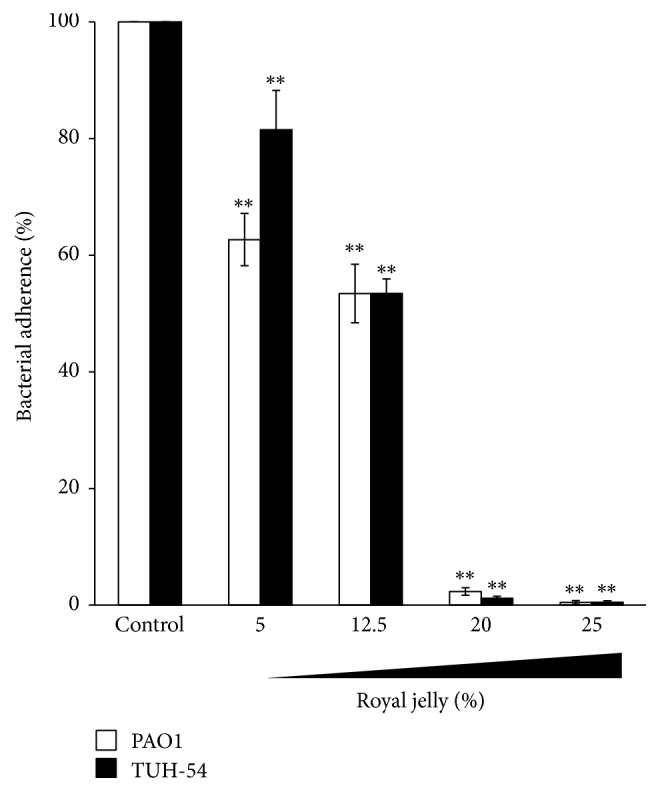
The inhibition of royal jelly to* P. aeruginosa* bacterial adherence. The inhibitory effect of royal jelly to the adherence of* P*.* aeruginosa* PAO1 and TUH-54 on abiotic surface. Data represent the means ± SDs of 4 independent experiments. Asterisks indicate significant differences (^*∗∗*^*p* < 0.001) versus control group (0% royal jelly).

The results of microtiter plate biofilm assay demonstrated that 25% royal jelly almost completely inhibited the bacterial adherence in PAO1, TUH-54, TUH-124, TUH-190, and TUH-213 (99-100% inhibition, data not shown). Furthermore, in order to determine the effective concentration of royal jelly to inhibit bacterial adherence, we performed dose-dependent experiments.  Figure 1 shows that royal jelly could inhibit bacterial adherence at the concentration of 5% to both PAO1 and representative isolate, TUH-54. This inhibitory effect was increased as higher concentration of royal jelly. These results suggest that royal jelly has the potential to inhibit biofilm formation of* P. aeruginosa* by the inhibition of initial attachment on abiotic surface, such as medical devices. The ability of royal jelly to inhibit the attachment of bacteria closely related to its antibacterial components. Among the components of royal jelly including sugar proteins and lipids, it has been proven that glycoproteins Jelleine-I–III have antibacterial activity against both Gram-positive and Gram-negative bacteria [16].* P. aeruginosa* virulence factor, Lectin B, which functions as the bacterial adhesin, could be blocked by royal jelly so that this bacterium cannot attach to the substrate [17].

**Figure 2:**
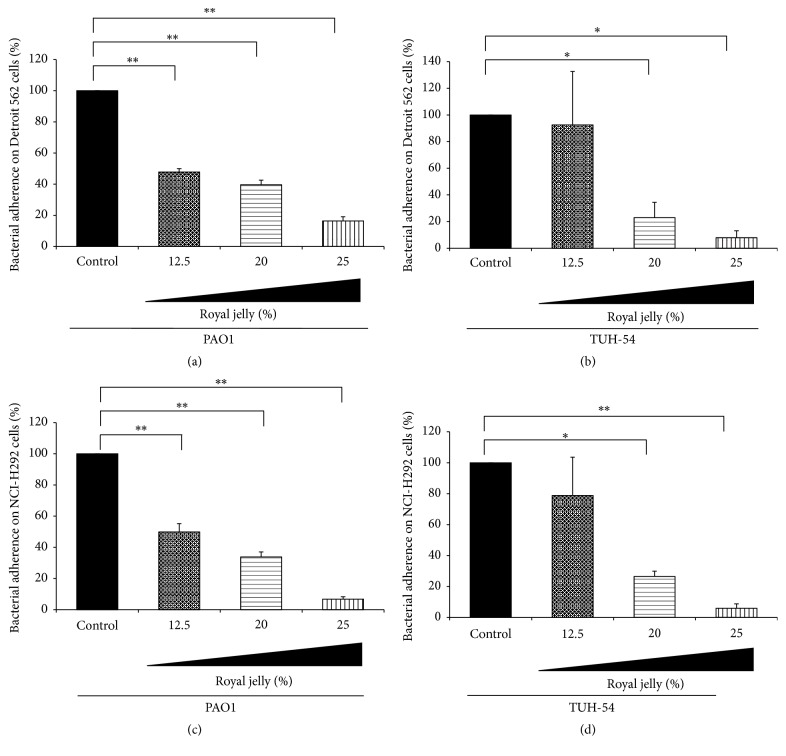
The inhibitory effect of royal jelly on the adherence of PAO1 and TUH-54 on human pharyngeal and lung epithelial cells. The inhibitory effect of royal jelly on the adherence of PAO1 (a, c) and TUH-54 (b, d) to Detroit 562 (a, b) and NCI-H292 (c, d) cells. Data represent the means ± SDs of 4 independent experiments. Asterisks indicate significant differences (^*∗∗*^*p* < 0.001 and ^*∗*^*p* < 0.01) versus control group (0% royal jelly).

In addition to the adherence on abiotic surface, we next examine whether the royal jelly has the ability to inhibit the adhesion of* P. aeruginosa* to the epithelial cells. The bacterial adherence assay on epithelium cells demonstrated that the adherence of both PAO1 and the representative clinical isolate, TUH-54, was inhibited by 30 min pretreatment with various concentrations of royal jelly (Figure 2).  Figure 2(a) shows that the adherence of PAO1 on Detroit 562 cells was inhibited by pretreatment of royal jelly in dose-dependent manner. The adherence of TUH-54 on the same cells was also significantly inhibited in 20% and 25% royal jelly-treated groups (Figure 2(b)). Furthermore, the effect of royal jelly on the adherence of PAO1 and TUH-54 to NCI-H292 cells showed similar results to those on Detroit 562 cell (Figures 2(c) and 2(d)).

Bacterial adhesion is an initial step in the pathogenic mechanism of* P. aeruginosa* infection. Oligosaccharide-mediated bacterium-cell recognition and adhesion are crucial for their colonization. Lectins play important role in glyconic recognition pattern as adhesins. There are two soluble lectins, Lec-A (PA-IL) and Lec-B (PA-IIL), in* P. aeruginosa*. These lectins are present on bacterial outer membrane and bind to galactose and fucose, respectively. They contribute important role in tissue damage caused by* P. aeruginosa* [18–21]. Some inventions have found the possibility of using specific lectins inhibitor as an alternative way to control the growth of* P. aeruginosa* and to reduce lung injury and as well as mortality caused by the same case, and therefore, lectins inhibition by specific carbohydrates proposed new perspective in therapy of* P. aeruginosa* infection [21]. Previous studies have confirmed that LecB binds to royal jelly and its hemagglutination activity was also inhibited by this compound [17]. Besides LecB, there are other adheren components, such as flagella and type IV pili, which are virulence factors and play a role in the attachment of bacteria to lung epithelial cells [22]. The bind between the adhesins with a membrane receptor initiates bacterial attachment to the epithelial cells [22]. Apart from the important role of flagella and pili, lectins, LecA and LecB, recently become a new target in the development of new antibacterials [23]. So far, royal jelly is known to inhibit the activity of LecB, but its effect on flagella and pili is still unknown. The findings from this study showed the potential inhibitory effect of royal jelly on adherence of* P. aeruginosa* to the human pharyngeal and lung epithelial cells and provide valuable information for the management of* P. aeruginosa* infections in clinical work since bacterial adhesion and biofilm formation play important roles in the initial bacterial infection. These results are also supported by previous study using antiadhesion-active components of edible seeds [24]. Siryaporn et al. showed that virulence activated through surface attachment required quorum sensing (QS) system (mediated by LasR) [25]. However, after 16 h incubation with 25% royal jelly, the expressions of* lasR* gene were not affected by royal jelly using qRT-PCR, suggesting that royal jelly has no effect on QS system (Supplemental Figure in Supplementary Material available online at https://doi.org/10.1155/2017/3191752). The action mechanisms of royal jelly for the inhibition of* P. aeruginosa* adherence are unknown and then the study regarding the influence of royal jelly on various adhesins of* P*.* aeruginosa*, such as LecB, flagellin, and pili, to epithelial cells is now under investigation.

### 3.3. Cytotoxicity and Protective Effect of Royal Jelly on Epithelial Cells

**Figure 3:**
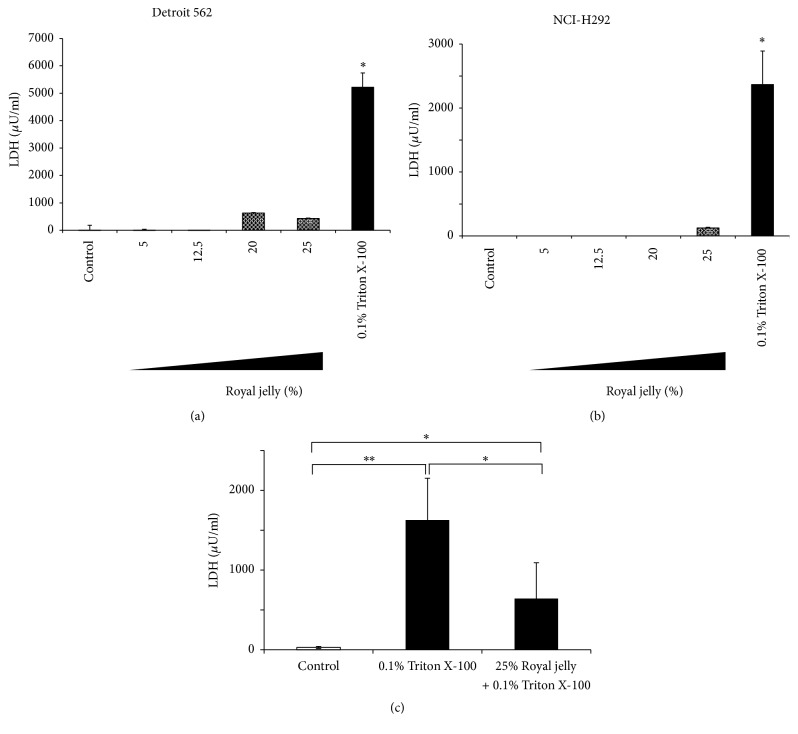
No cytotoxicity effect of royal jelly extract on pharyngeal, Detroit 562 (a) and lung and NCI-H292 (b), epithelial cells. As a positive control, epithelial cells were treated with 0.1% Triton X-100 and shaken gently for 10 min at room temperature. Epithelial protective effect of royal jelly against 0.1% Triton X-100 (c). Detroit 562 monolayers cultured in a 24-well plate were pretreated with 25% royal jelly or PBS for 30 s at room temperature, washed with PBS twice, and irritated with 0.1% Triton X-100 for 30 sec at room temperature. Data represent the means ± SDs of 3 independent experiments. Asterisks indicate significant differences (^*∗∗*^*p* < 0.001 and ^*∗*^*p* < 0.01) versus control group (0% royal jelly).

To confirm the absence of the effect of royal jelly on human pharyngeal and lung epithelial cells, we measured the level of LDH released from two epithelial cell lines, Detroit 562 cells and NCI-H292.  Figure 3 shows that there are no significant differences in released LDH activity even after treatment with 25% royal jelly. Royal jelly is one of nutritional supplements that are safe and widely consumed. Giving royal jelly orally, as much as 10 g kg^−1^, did not cause acute cytotoxic reactions in rats [26]. Our data also support the safety of royal jelly to use for pharynx and lung epithelial cells.

To evaluate the effect of royal jelly for preventing cell damage, LDH assay was performed by the addition of 0.1% Triton X-100 after pretreatment with 25% royal jelly. As shown in Figure 3(c), royal jelly reduced LDH release by Triton X, suggesting that royal jelly has physically protective effect for epithelial cells.

### 3.4. Royal Jelly Inhibits Overproduction of Chemokines in Human Pharyngeal and Lung Epithelial Cells

**Figure 4:**
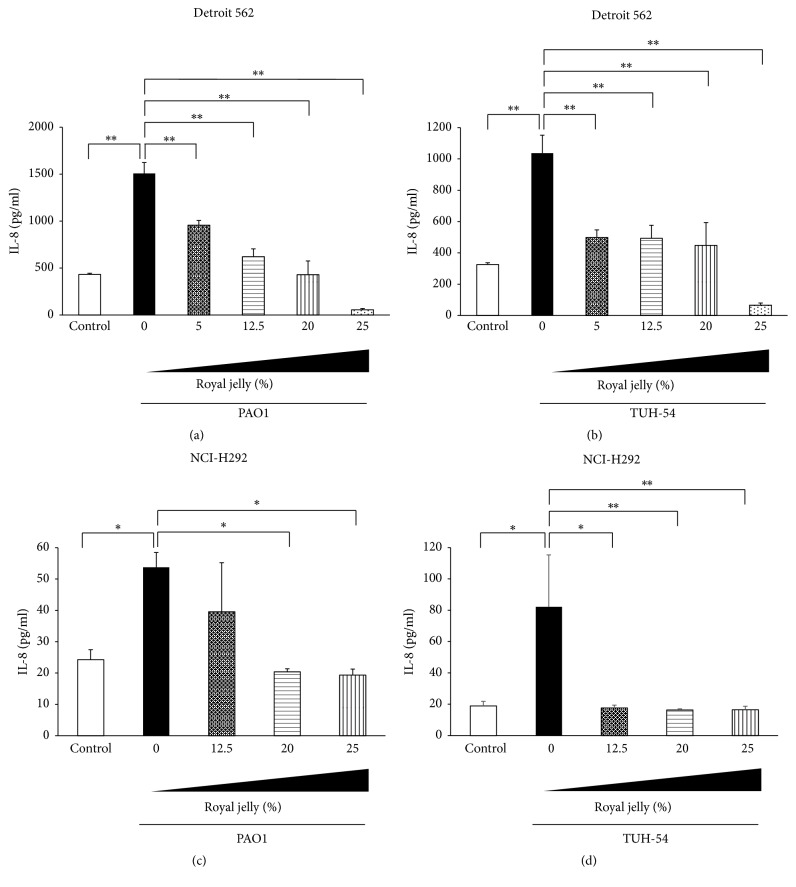
Inhibitory effect of royal jelly on IL-8 overproduction in human pharyngeal, Detroit 562 (a, b) and lung and NCI-H292 (c, d), epithelial cells stimulated with* P*.* aeruginosa* PAO1 (a, c) and TUH-54 (b, d) for 4 h. Data represent the means ± SDs of 4 independent experiments. Asterisks indicate significant differences (^*∗∗*^*p* < 0.001 and ^*∗*^*p* < 0.01) between the indicated groups.

**Figure 5:**
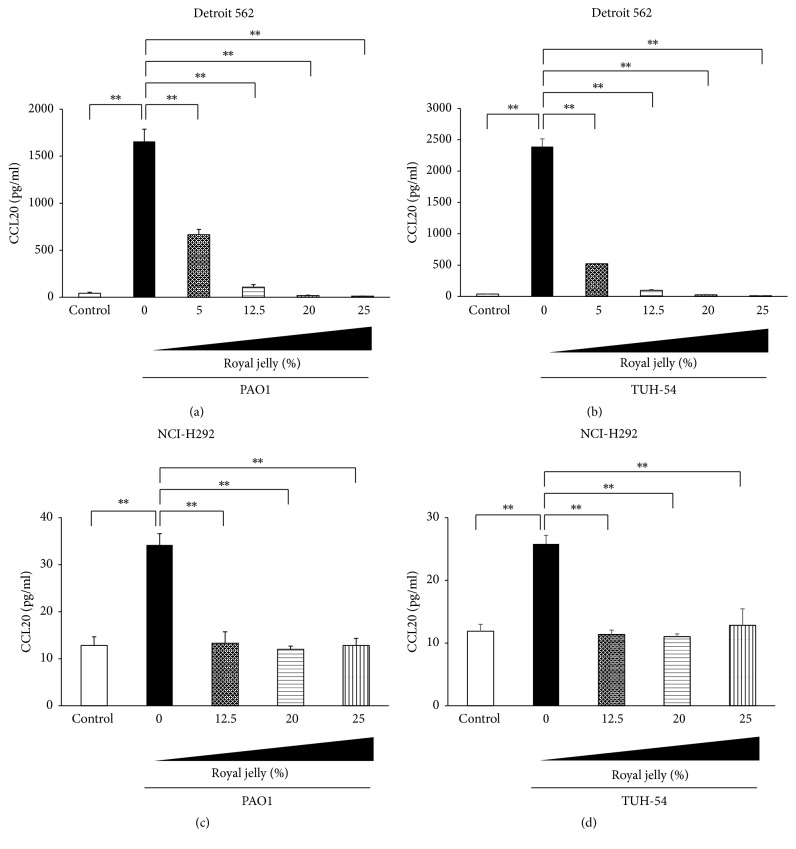
Inhibitory effect of royal jelly on CCL20 overproduction in human pharyngeal, Detroit 562 (a, b) and lung and NCI-H292 (c, d), epithelial cells stimulated with* P*.* aeruginosa* PAO1 (a, c) and TUH-54 (b, d) for 4 h. Data represent the means ± SDs of 4 independent experiments. Asterisks indicate significant differences (^*∗∗*^*p* < 0.001) between the indicated groups.

We next investigated the preventive effects of pretreatment with royal jelly on chemokines productions in* P*.* aeruginosa* PAO1 or TUH-54-stimulated human pharyngeal and lung epithelial cells.* P*.* aeruginosa* PAO1 and TUH-54 strains significantly induced IL-8 and CCL20 productions in Detroit 562 and NCI-H292 cells after 4 h incubation (Figures 4 and 5). Figures 4(a) and 4(b) show that the IL-8 overproduction in pharyngeal epithelial cells, Detroit 562, after the stimulation with PAO1 and TUH-54 strains was significantly reduced by the 30 min pretreatment with royal jelly in dose-dependent manner. Moreover, the pretreatment with 20% and 25% royal jelly significantly inhibited IL-8 production in PAO1-stimulated NCI-H292 cells (Figure 4(c)) and more than 12.5% royal jelly significantly reduced IL-8 production in TUH-54-stimulated NCI-H292 cells (Figure 4(d)). It is interesting that only 30 min pretreatment of epithelial cells is effective for inhibition of IL-8 overproduction by* P*.* aeruginosa* infection.

Figure 5 also shows the inhibitory effect of 30 min pretreatment with royal jelly on CCL20 overproduction in human pharyngeal and lung epithelial cells after stimulation with PAO1 or TUH-54 strain for 4 h. This inhibitory effect on CCL20 production was similar to that on IL-8.

**Figure 6:**
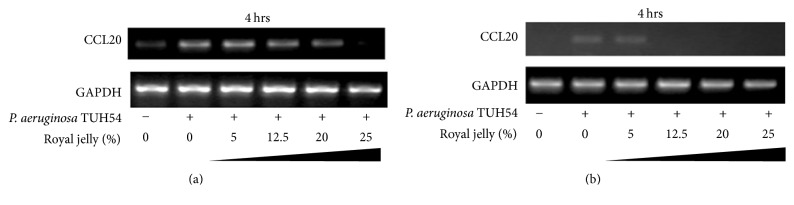
The inhibitory effect of royal jelly on CCL20 mRNA expression in human pharyngeal, Detroit 562 (a) and lung and NCI-H292 (b), epithelial cells stimulated with* P*.* aeruginosa* TUH-54 strain for 4 h.

We finally confirmed the mRNA expression levels of CCL20 and IL-8 in human pharyngeal and lung epithelial cells stimulated with* P*.* aeruginosa* TUH-54 or PAO1 for 4 h by RT-PCR.  Figure 6 shows that mRNA expression level of CCL20 in* P*.* aeruginosa* TUH-54-stimulated Detroit 562 and NCI-H292 cells for 4 h was inhibited by 30 min pretreatment with more than 12.5% royal jelly in both epithelial cells. Similar results were seen in the mRNA expression of IL-8 in both epithelial cells exposed to PAO1 and TUH-54 strains (data not shown). These results are consistent and supported by previous study showing that royal jelly has anti-inflammatory effects to suppress LPS-induced IL-6 and CXC chemokine ligand 10 from the periodontal ligament cell line [27].

IL-8 is a proinflammatory cytokine that can be induced in the event of bacterial infections and has a key role in the recruitment and activity of neutrophils, which are considered major contributors to the tissue damage during inflammatory diseases [28–30]. The production of this molecule is increased in cystic fibrosis mouse model infected with* P. aeruginosa* LPS and bacterial culture supernatant [31]. The involvement of* P. aeruginosa* in the oropharyngeal infection is very important to consider. Other studies also proved that LPS and bacterial culture supernatant induce the production of IL-8 [32] and human bronchial epithelial cells increased the production of IL-8 by 5 h exposure with* P. aeruginosa* LPS [33]. Regarding novel mechanism involved in this induction, it has been suggested that the outer membrane vesicle, which is common in all Gram-negative bacteria, plays an important role to deliver short RNA into host cells [33]. CCL20 has been shown to act as a chemotactic factor that attracts strongly lymphocytes and slightly neutrophils into inflammatory lesions [34]. This study demonstrated that royal jelly can inhibit the increased production of IL-8 and CCL20 by stimulation with* P. aeruginosa* and these interesting findings encourage us to clarify this inhibitory mechanism. Collectively, our results suggest that royal jelly can reduce the inflammatory response against* P. aeruginosa* infection in pharyngeal and lung epithelial cells and may be a useful supplement as complementary and alternative medicine for preventing respiratory infection caused by* P. aeruginosa*.

## 4. Conclusions

Our results demonstrated that royal jelly can inhibit the adherence of* P*.* aeruginosa* PAO1 and TUH-54 on abiotic surface in a dose-dependent manner. In addition, we elucidated that 30 min pretreatment with royal jelly can inhibit the adhesion of both* P*.* aeruginosa* strains in pharynx and lung and significantly reduce the overproduction of IL-8 and CCL20 in both* P. aeruginosa*-stimulated epithelial cells. Royal jelly could suppress the production of proinflammatory cytokines by inhibiting the adherence of bacteria to epithelial cells. Furthermore, these findings suggest that certain components of royal jelly, when identified and studied in much more detail, might be used as complementary medicine.

## Supplementary Material

No inhibitory effect of royal jelly on lasR gene expression in *P. aeruginosa* PAO1 and TUH-54. Data represent the means ± SDs of 3 independent experiments.

## Figures and Tables

**Table 1:** Susceptibility to antibiotics and royal jelly for *P. aeruginosa*.

Strain	Origin	MIC (*μ*g mL^−1^)			MIC (%)
		IPM	CPFX	AMK	Royal jelly
PAO1	Wild type	1	0.25	2	≥50
TUH-54	Pharyngeal secretions	8	0.5	16	≥50
TUH-122	Coughed-up sputum	2	0.5	4	≥50
TUH-188	Oral abscess	2	0.125	4	≥50
TUH-213	Oral abscess	2	0.125	4	≥50
