# Supplementary material for: Royal Jelly Inhibits Pseudomonas aeruginosa Adherence and Reduces Excessive Inflammatory Responses in Human Epithelial Cells
Source: Biomed Res Int. 2017 Sep 17;2017:3191752. doi: 10.1155/2017/3191752 (PMC5623779; doi:10.1155/2017/3191752)
Supplement: Supplementary file 1 — No inhibitory effect of royal jelly on lasR gene expression in P. aeruginosa PAO1 and TUH-54. Data represent the means ± SDs of 3 independent experiments. [file 3191752.f1.docx]

**Supplemental Methods**

**Gene expression of *lasR* genes.** PAO1 and TUH54 were grown were incubated in the presence or absence of 25% royal jelly in LB medium. After 16-h incubation, total RNA was isolated using an RNeasy Mini kit (QIAGEN, Valencia, CA, USA). Transcriptor First Strand cDNA Synthesis kit (Roche Diagnosis, Mannheim, Germany) was used for reverse transcription. qRT-PCR was performed using StepOnePlus^TM^ Real Time PCR System with Fast SYBR^®^ Green Master Mix (Thermo Fisher Scientific, MA, USA). The expression level of *lasR* gene was normalized to the expression of *rpsL* whose expression remained constant throughout the experiment. The threshold cycle values and data analyses were performed by StepOne™ Software v2.2 (Thermo Fisher Scientific). The results were expressed as fold-change values relative to the control samples. The sequences for qRT-PCR were as follows;

lasR-F: TTTCTGGGAACCGTCCATCT, lasR-R: GCCGAGGCTTCCTCGAA, rpsL-F: GCAACTATCAACCAGCTGGTG, rpsL-R: GCTGTGCTCTTGCAGGTTGTG.

**Supplemental Figure. Heni S. et al.**

**Relative *lasR* transcription / Control**

control control

25% Royal Jelly 25% Royal Jelly

TUH-54

PAO1
